# Autoimmunity in thymic epithelial tumors: a not yet clarified pathologic paradigm associated with several unmet clinical needs

**DOI:** 10.3389/fimmu.2024.1288045

**Published:** 2024-04-02

**Authors:** Matteo Perrino, Emanuele Voulaz, Simone Balin, Gerardo Cazzato, Elena Fontana, Sara Franzese, Martina Defendi, Fabio De Vincenzo, Nadia Cordua, Roberto Tamma, Federica Borea, Marta Aliprandi, Marco Airoldi, Luigi Giovanni Cecchi, Roberta Fazio, Marco Alloisio, Giuseppe Marulli, Armando Santoro, Luca Di Tommaso, Giuseppe Ingravallo, Laura Russo, Giorgio Da Rin, Anna Villa, Silvia Della Bella, Paolo Andrea Zucali, Domenico Mavilio

**Affiliations:** ^1^ Department of Medical Oncology and Hematology, IRCCS Humanitas Research Hospital, Milan, Italy; ^2^ Division of Thoracic Surgery, IRCCS Humanitas Research Hospital, Milan, Italy; ^3^ Department of Biomedical Sciences, Humanitas University, Milan, Italy; ^4^ Department of Medical Biotechnologies and Translational Medicine, University of Milan, Milan, Italy; ^5^ Section of Pathology, Department of Precision and Regenerative Medicine and Ionian Area (DiMePRe-J), University of Bari “Aldo Moro”, Bari, Italy; ^6^ Istituto di Ricerca Genetica e Biomedica (IRGB), National Research Council (CNR), Milan, Italy; ^7^ Human Genome and Biomedical Technologies Unit, IRCCS Humanitas Research Hospital, Milan, Italy; ^8^ Unit of Clinical and Experimental Immunology, IRCCS Humanitas Research Hospital, Milan, Italy; ^9^ Section of Human Anatomy and Histology, Department of Translational Biomedicine and Neurosciences (DiBraiN), University of Bari “Aldo Moro”, Bari, Italy; ^10^ Department of Pathology, IRCCS Humanitas Research Hospital, Milan, Italy; ^11^ Clinical Laboratory, IRCCS Humanitas Research Hospital, Milan, Italy; ^12^ San Raffaele Telethon Institute for Gene Therapy (SR-TIGET), IRCCS San Raffaele Scientific Institute, Milan, Italy

**Keywords:** thymic epithelial tumors, autoimmunity, myasthenia gravis, immunotherapy, surgery, thymopoiesis

## Abstract

Thymic epithelial tumors (TETs) are rare mediastinal cancers originating from the thymus, classified in two main histotypes: thymoma and thymic carcinoma (TC). TETs affect a primary lymphoid organ playing a critical role in keeping T-cell homeostasis and ensuring an adequate immunological tolerance against “self”. In particular, thymomas and not TC are frequently associated with autoimmune diseases (ADs), with Myasthenia Gravis being the most common AD present in 30% of patients with thymoma. This comorbidity, in addition to negatively affecting the quality and duration of patients’ life, reduces the spectrum of the available therapeutic options. Indeed, the presence of autoimmunity represents an exclusion criteria for the administration of the newest immunotherapeutic treatments with checkpoint inhibitors. The pathophysiological correlation between TETs and autoimmunity remains a mystery. Several studies have demonstrated the presence of a residual and active thymopoiesis in adult patients affected by thymomas, especially in mixed and lymphocytic-rich thymomas, currently known as type AB and B thymomas. The aim of this review is to provide the state of art in regard to the histological features of the different TET histotype, to the role of the different immune cells infiltrating tumor microenvironments and their impact in the break of central immunologic thymic tolerance in thymomas. We discuss here both cellular and molecular immunologic mechanisms inducing the onset of autoimmunity in TETs, limiting the portfolio of therapeutic strategies against TETs and greatly impacting the prognosis of associated autoimmune diseases.

## 1 Introduction

The immune system comprises an intricate system of cells, tissues, and organs that collaborate to safeguard the body against infections and disease. In addition to providing protection from infection, the immune system has a significant impact on the initiation and advancement of cancer. The relationship between the immune system and cancer is complex and not fully understood. However, research has shown that the immune system can both promote and suppress the growth of cancer cells. In certain instances, the immune system is capable of identifying and eliminating cancer cells, whereas in other cases, it can help cancer cells to grow and spread. The immune system can also be used to treat cancer. Immunotherapy is a kind of treatment that uses the patient immune system to control the development of cancer. This type of therapy can be used to stimulate the immune system to recognize and hit cancer cells, or to block signals that help cancer cells to grow and spread. When the immune system erroneously targets and damages normal cells and tissues within the body, it generates autoimmune diseases. In some cases, autoimmune diseases can cause the immune system to become overactive, leading to an increased risk of developing certain types of cancer.

The thymus is the lymphoid organ deputed to the maturation and selection of T cells, and the induction of central T cell tolerance. Thymic epithelial tumors (TETs) originate from the thymus gland and represent the most common neoplasms of the anterior mediastinum. Furthermore, TETs, are frequently associated with autoimmune diseases (ADs). TETs are rare tumors, with an incidence of 1.3-3.2 cases per million/year (1). They are typically diagnosed in the fifth and sixth decades of age and are more common in males. According to The World Health Organization (WHO), TETs are divided in Thimoma A, AB, B1, B2, B3 and thymic carcinoma (TC) (2). Compared to thymomas, which have a higher incidence and more favorable characteristics, TCs are very rare and show a higher tendency to lymphatic and blood spread causing a worse prognosis. The American Joint Committee on Cancer (AJCC) 8th Edition of clinical TNM staging classified TETs from stage I to stage IVb based on the level of local invasion, lymph node involvement and distant metastasis (3).

Management of TETs is still quite arduous: most of clinical evidence is based on retrospective analyses, prospective single arm trials, and experts’ opinion. Surgery remains today the main treatment for patients with resectable thymoma. Nevertheless 10–30% of patients can relapse even after 10 to 20 years from radical surgery (4–11). The majority of recurrences are locoregional (mostly in pleura, pericardium, and mediastinum), while distant spread is rare (12). In case of no-resectable local advanced or metastatic disease, the current state of art is platinum-based chemotherapy regimens: cisplatin/anthracyclines (CAP or ADOC) or etoposide for thymoma and carboplatin/paclitaxel for TC (13). However, chemotherapy has modest efficacy in the palliative setting, and there is no clear evidence and consensus on options for progression after first line systemic therapy (13). The introduction of phase II and III clinical trials is challenging due to the low incidence of TETs, and the process of discovering new therapeutic targets is slow. Promising results have been obtained in stage IV diseases with anti-angiogenic agents (Sunitinib and Lenvatinib), cKIT inhibitors (Imatinib), mTOR inhibitors (Everolimus) and immunotherapy with anti PD-1 inhibitors (14–20). PD-L1 is an immune-inhibitory molecule that, binding its receptor (PD1) on immune cells, modulates the activity of the immune system, suppressing the activation of T cells and thus promoting tumor escape from the immune control and subsequent tumor progression. PD-L1 is overexpressed in several solid tumor types. The block of PD-L1/PD-1 interaction through immune check points inhibitors (ICIs) has shown interesting results, and currently PD-1/PD-L1 inhibitors are a standard of care in many malignances (21, 22). The expression of PD-L1 in thymomas ranges from 23% to 92%, providing a rationale for the use of PD-1/PD-L1 inhibitors for the treatment of these tumors (13). Nevertheless, the association between thymic neoplasia, in particular thymoma, and ADs justifies a higher degree of immune related adverse events (IRAEs) that should be carefully taken into account (23).

In fact, TETs develop in the lymphoid organ playing a key role in keeping T cell homeostasis and ensuring an optimal degree of immunologic tolerance against “self”. This process is highly active in the first years of life and fades with aging when thymus becomes highly atrophic and senescent. However, TETs are not commonly diagnose in children and young adults, with their incidence becoming more frequent in middle age and peaking during the sixth and seventh decades of life. Moreover, Thymoma (but not TC) is associated with the onset of Myasthenia Gravis (MG) and other ADs. These co-morbidities represent a limit for the application of novel immunotherapies that are not administered to patients with thymomas to avoid the higher risk of either inducing or exacerbating autoimmunity.

Taken together, this clinical evidence renders TETs one of the most interesting disease models at the intersection between tumor-immunology and autoimmunity. This review aims to report the currently available evidence on TETs and systemic autoimmune diseases. In particular, we reviewed the literature data regarding ADs affecting patients with TETs, the role of thymus in the generation of T cells and control of the immune system, the contribution of immune cells in TETs and associated autoimmunity, the correlation between histotype and tumor microenvironment in thymic neoplasms and the role of immunotherapy.

## 2 Autoimmune diseases and thymic epithelial tumors

The thymus is the primary lymphoid organ deputed to the maturation and selection of T cells and the induction of central T cell tolerance. Hence, it is not surprising that thymomas are associated with a variety of autoimmune disorders (**Table 1**) (24–26). The most common TET-associated AD is Myasthenia Gravis (MG), present in 30% of patients affected by thymoma.

**Table 1:** Autoimmune diseases associated with Thymoma [adapted from Marx et al. (24)].

Autoimmune disease	Frequency	Associated WHO Thymoma Type
Myasthenia Gravis	30%-40%	B1, B2, B3>AB>A
Neuromyotonia (Isaac’s Syndrome)	≈3%	B2, B3, AB, TC
Encephalitis	<1%	A, B1, B2, B3
Polymyositis	1%-5%	B1, B2
Good’s syndrome	5%-20%	B2>AB, B1, B3>A
Pure red cell aplasia	≈4%	AB>B2, B1>A, B3
Systemic Lupus Erythematosus	≈2%	B2>AB, A
Rheumathoid arthritis	<1%	B1,B3
Sjogren’s syndrome	<1%	B1

The main clinical manifestation of MG is fatigable muscle, weakness, and the symptoms vary depending on the muscle district involved. Other symptoms that may raise suspicion of MG are dysphagia, dysarthria, diplopia and asymmetric eyelid ptosis (bulbar symptoms).

Pathogenic antibodies detected in MG bind to extracellular determinants of post-synaptic membrane proteins and cause morphological and functional changes that alter neuromuscular transmission. Anti-acetylcholine receptor antibodies (AChR-Abs) are present in approximately 90% of patients with MG, while the remaining 10% of patients have muscle-specific anti-tyrosine kinase antibodies (Musk-Abs) in 25-47% of cases (27).

The diagnosis of MG can be confirmed not only by antibody tests but also by electromyographic studies that reveal a deficit in post-synaptic neuromuscular transmission (28). MG therapy is modulated in relation to the severity of the symptoms: pyridostigmine is the most used drug and it can be variably associated with corticosteroids and immunosuppressants such as ciclosporin, azathioprine and mycophenolate mofetil. Intravenous immunoglobulin administration and plasmapheresis are indicated for more severe exacerbations of the disease (29). For patients with MG associated with resectable thymoma, radical thymectomy represents the standard of care, but pharmacological therapy is still useful to control symptoms and avoid worsening of possible respiratory failure (26, 30). Patients with one autoimmune disease often have other immune-mediated conditions; approximately 10%-15% of patients affected by thymoma present paraneoplastic disorders other than MG (31).

Limbic encephalitis is a paraneoplastic neurological disease that usually presents with memory loss, confusion, changes in behavior or mood, seizures and hallucinations. Patients with thymoma-associated paraneoplastic limbic encephalitis often have elevated levels of antineuronal nuclear antibodies type 1, Ma2 antibodies, or collapsing response-mediating protein-5 antibodies (32).

Acquired neuromyotonia, also known as Isaac’s syndrome, is a disease that results from the hyperexcitability of peripheral nerves conditioning continuous activity of muscle fibers. It usually presents with muscle stiffness, cramps and myoclonia. Approximately 38-50% of patients with Isaac syndrome have autoantibodies against presynaptic voltage-gated potassium channels; in 20% of cases a thymoma is associated (33).

Polymyositis is another inflammatory myopathy frequently related to thymoma and MG. Also in this case the typical symptomatology consists of proximal muscle weakness. It is usually associated with elevated levels of creatine kinase and the presence of myositis-specific autoantibodies (34).

Other autoimmune conditions associated with TETs are autoimmune hepatitis and myocarditis; hematological diseases, such as pure red blood cell aplasia and T-cell lymphocytosis, can also be observed in patients affected by thymoma and polymyositis (35). The co-presence of thymoma and systemic lupus erythematosus (SLE) varies between 1.5% and 2% in epidemiological studies and approximately 2-10% of patients affected by thymoma develops SLE manifesting the classic symptoms with serositis and skin and articular involvement (36–38).

A special mention should be deserved to Good’s syndrome (GS), a rare association of thymoma, hypogammaglobulinemia, and marked reduction of peripheral B cells. It occurs in 5% of patients with thymoma and has a highly variable clinical presentation, consisting of a spectrum of recurrent invasive bacterial and opportunistic infections, and occurrence of autoimmune manifestations in more than 50% of cases (39–41). It has been suggested that autoreactive cytotoxic T cells derived from aberrant T cell maturation in the thymic tumor microenvironment may be involved in B cell lymphopenia (41), but the pathogenesis of GS is largely unknown. A recent study by Torres-Valle and colleagues demonstrated that immune defects in GS patients involve not only B and T cells, but also several populations of innate immune cells (42), suggesting that GS may represent an interesting model to investigate the immune dysregulation mechanisms underlying the association between autoimmunity and thymic epithelial tumors.

## 3 The role of thymus in the generation of T cells and control of the immune system

**Figure 1:**
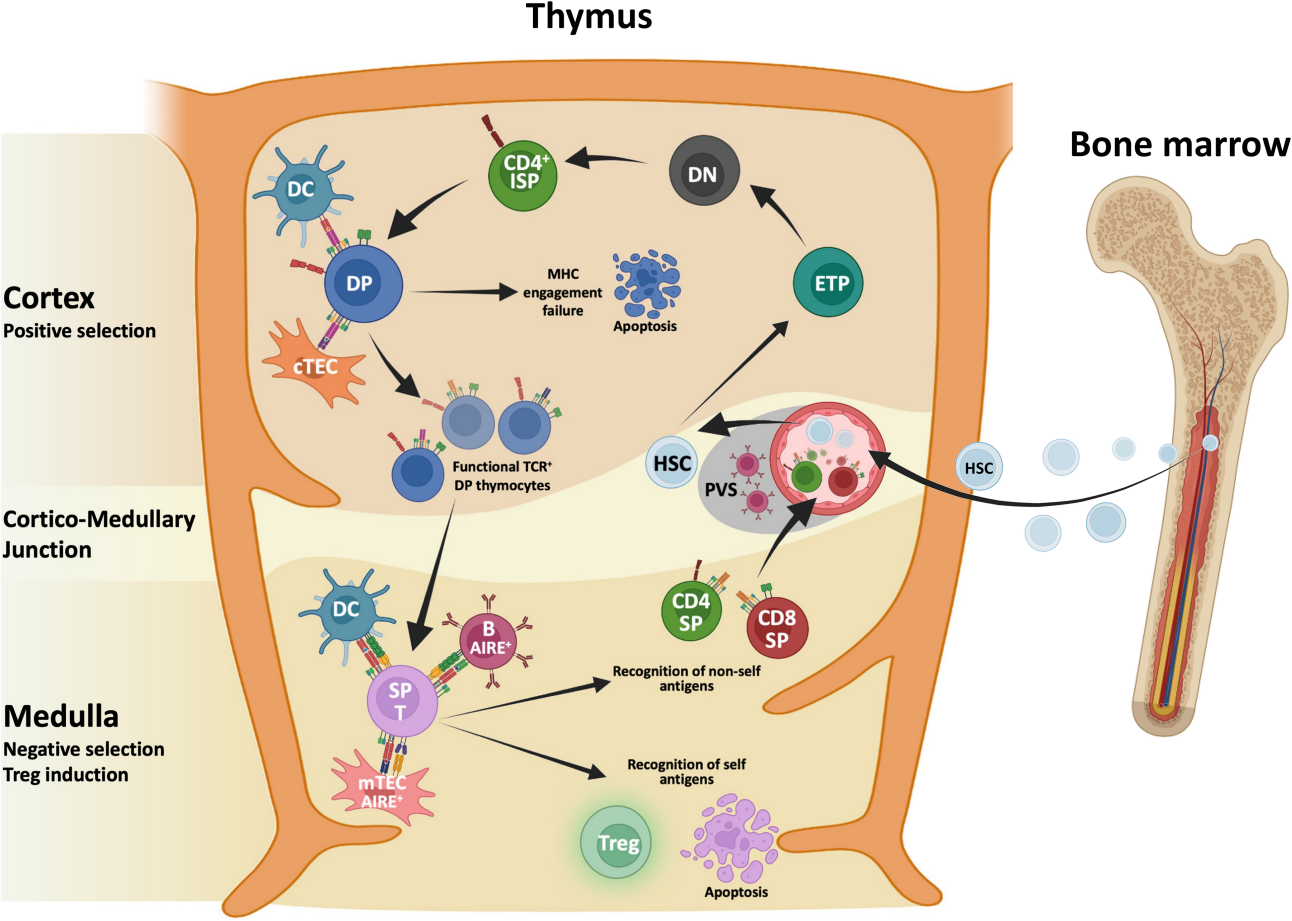
Schematic representation of T cell development in normal thymus. Hematopoietic stem cells (HSC) deriving from the bone marrow seed the thymus and progress toward early thymocyte precursors (ETPs). ETPs moving within the cortex differentiate toward the double negative (DN) stage and before becoming double positive (DP) thymocytes, this DN-DP transition involves an immature single positive (ISP) intermediate characterized by the expression of the co-receptor CD4 and by the lack of CD3. Thymocytes interacting with major histocompatibility complex (MHC) molecules expressed by cortical thymus epithelial cells (cTECs) and dendritic cells (DCs) are positively selected. Thymocytes that do not recognize MHC molecules die for the loss of survival signals. Surviving thymocytes upregulate either CD4 or CD8 and migrate toward the medulla where negative selection, mediated by medullaryTECs (mTECs), DCs and a subset of B cells, induce the deletion of auto reactive thymocytes. A small fraction of thymocytes that show intermediate to high-affinity TCR that binds self-antigens and a high expression of CD25 escape clonal deletion becoming regulatory T cells (Treg). After completing the maturation CD4 or CD8 single-positive (SP) T cells leave the thymus, as recent thymic emigrants.

T cell differentiation and generation of a diversified repertoire of mature T cells reacting towards foreign antigens, while discriminating between self and non-self-antigens occurs in thymus, a specialized organ composed of various cells deriving from different embryonic germ layers (43). **Figure 1** illustrates T cell development in normal thymus. Thymocytes are the major cellular component together with other cells of the immune system including B cells, dendritic cells (DCs), macrophages, natural killer (NK) cells and iNKT cells. Immune cells interact with Thymic Epithelial Cells (TECs) that are divided in cortical TECs (cTECs) and medullary TECs (mTECs), each type playing different functions and having different spatial distribution (44). The crosstalk between TECs and nascent thymocytes is crucial for T cell differentiation and maturation along with the establishment of “self-nonself discrimination” mechanisms aimed at the elimination of autoreactive T cells carrying self-reactive T cell antigen receptor (TCR) or diverting into thymic regulatory T cells (45, 46).

During thymic journey, thymocytes encounter various cellular components including mesenchymal (pericytes and fibroblasts) and endothelial cells that contribute to shape their differentiation and maturation (47–49). Early thymocyte precursors (ETPs) derived from the bone marrow seed the thymus and get in touch with TECs and other cellular components. The mutual interplay of ETPs with various thymic components modulate differentiation, survival, and lymphocyte selection. Cortical compartment development strictly depends on ETP homing, while mTEC compartment induction relies on thymocyte-derived signals including RANK, CD40 and Lymphotoxin Beta (50–52). During thymic journey, ETPs move from the cortex undergoing various maturation stages starting with CD4^-^/CD8^-^ double negative (DN) cells, progressing towards immature single-positive thymocytes expressing CD4, but not CD8 (CD4ISP) before differentiating into double positive (DP) thymocytes. TCR rearrangement occurs during T cell differentiation progression, and once successful rearrangement of TCRα and TCRβ loci are completed, nascent thymocytes undergo positive and negative selection processes that result in the elimination of self-reactive T cells (53). Thymocytes interacting with self Major Histocompatibility Complex (MHC) molecules expressed by cTECs are positively selected in the cortex. A first wave of negative selection mediated by DCs occurs in the cortex to eliminate autoreactive thymocytes. Surviving thymocytes differentiate into single positive (SP) CD4^+^ and CD8^+^ cells that migrate to the medulla where they undergo negative selection mediated by mTECs, a heterogenous population that include immature and CCL21^+^ mature mTEC^low^, AIRE^+^ mTEC^high^, corneocyte-like mTECs and tuft cell-like mTECs (54–58). The mTEC^high^ subpopulation expresses the transcriptional activator Autoimmune Regulator Element (AIRE) that allows the expression of tissue restricted antigens (TRAs), which are subsequently presented on MHC molecules to SP thymocytes. Single cell RNA seq analysis revealed that mTECs express over 18,000 genes, encompassing about 85% of the protein-coding, and 4,000 of those regulated by AIRE (59–61). Recent evidence identifies AIRE as a chromatin binding molecule suggesting that AIRE may act as a coactivator rather functioning as a traditional transcription factor (62). Negative selection process leads to the elimination of thymocytes with high affinity for self-antigens while weaker interaction leads to the generation of organ-specific T regulatory (Treg) cells. This latter process needs AIRE-dependent expression of TRAs (63–66). Of note, other antigen presenting cells (APCs) have a role in mediating clonal deletion and Treg cell induction. Murine studies indicate that conventional DCs, thymic plasmacytoid and trans-endothelial DCs contribute to the presentation of peripheral antigens in the thymus. Finally, the thymic microenvironment hosts a population of B cells that contribute to antigen presentation. Importantly, a subpopulation of class-switched B cells expressing AIRE contribute to central tolerance, presenting some of the AIRE-dependent antigens to thymocytes (67). Taken together, these data along with recent findings of “misplaced” thymic stromal cells (68) highlight the complex interplay among various cellular players and lay the ground for a better comprehension of the impact of malignant transformation on stromal and epithelial remodeling that may impair the control of immune tolerance and favor the escape of tumor-associated thymocytes.

## 4 The role of immune cells in TETs and associated autoimmune diseases

### 4.1 T lymphocytes

TETs originate from TECs, and all subtypes of TETs except for TCs can retain, to a different extent, the capacity to promote T cell development. T cells within thymomas can be found at different stages of maturation including DN and DP thymocytes and the more mature CD4 and CD8 SP populations (69, 70). Indeed, several studies demonstrated that an active thymopoiesis occurs in thymomas (71–75). Of note, Inoue et al. (71) demonstrated the presence of ETPs, characterized by the expression of the hematopoietic stem cells marker CD34, within thymomas, thus suggesting the active recruitment of T cell precursors by these tumors. Furthermore, thymomas were also characterized by the accumulation of CD4ISP and DP thymocytes compared with healthy thymuses derived from pediatric patients who underwent corrective cardiac surgery. In particular, the mixed and lymphocytic-rich thymomas, currently known as type AB and type B thymomas, showed the highest frequency of these thymopoiesis intermediates (74, 75). DP thymocytes were also identified in thymic hyperplasia (TH), a benign lesion characterized by thymus enlargement. However, the accumulation of CD4ISP thymocytes was observed only in thymomas, suggesting that it may derive from either an inefficient positive selection or an increased recruitment of T cell progenitors only within malignant tumors (74, 75). Because the above-described studies were published before the more recent WHO classifications of thymomas that distinguish the different TET histotypes (2), the possible influence of specific thymoma subtypes on the thymopoiesis process was not addressed.

Another important issue emerging from these studies regards the fate of mature SP T cells. MG^-^associated thymomas (MG^+^ thymomas) have been reported to show a thymopoiesis skewed towards CD4^+^ T cells characterized by an increased intratumor frequency of naïve CD3^+^CD4^+^CD45RA^+^ T cells compared with thymomas not associated to MG (MG- thymomas) (76, 77). Notably, the increased frequency of intratumor CD4^+^ T cells was not associated with an increased frequency of circulating CD4^+^ RTE (73). On the contrary, in MG^-^ thymomas, a decreased intratumor CD4^+^/CD8^+^ ratio within the naïve T cell compartment was observed suggesting that thymopoiesis in MG^-^ thymomas is skewed toward CD8 rather than CD4 T lymphocyte commitment (77). Therefore, CD4^+^ lineage maturation appeared to be impaired only in MG- thymomas suggesting a possible role of CD4^+^ T lymphocytes, together with a defective thymocyte selection, in favoring AD onset (76).

Despite both thymomas and TCs arise from TECs, TCs are characterized by the lack of DP thymocytes, suggesting the absence of cortical functions and subsequent absence of ETP seeding and thymopoietic activity (74). Therefore, the role played by T cells in thymomas and TCs may differ.

In a recent study, Yamamoto et al. (78) assessed the anti-tumor activity of TET-associated CD4^+^ and CD8^+^ T cells. They observed that both intratumor CD4^+^ and CD8^+^ T cells of type B3 thymomas and TCs showed higher anti-tumor activity, assessed as cytokine production upon PMA/ionomycin stimulation, compared with the other thymoma subtypes. Moreover, type B3 thymomas and TCs were characterized by an increased frequency of CD4^+^ Treg, distinguished by high CD25 expression of CD25. Type B3 thymomas and TCs were also associated with an increased frequency of CD4^+^ and CD8^+^ T cells expressing the inhibitory receptors PD1 and TIM3. Notably, T lymphocytes isolated from these tumors showed enhanced *in vitro* anti-tumor cytotoxic activity upon treatment with anti-PD1 mAb, thus supporting a possible beneficial role of ICIs in unleashing the anti-tumor potential of T lymphocytes in type B3 thymomas and TCs.

### 4.2 B lymphocytes

Another player of adaptive immunity is represented by B lymphocytes, cells responsible for humoral immune responses (79). B cells are deputed to the production of antibodies; however, they are also involved in antigen presentation and play a relevant role in anti-tumor immunity. B cells can recognize and internalize antigens through their specific B Cell Receptor (BCR) and, upon processing, antigens bound to MHC class II molecules can be presented to T cells. The BCR specificity results in a completely different antigen repertoire that can be presented to T cells by B cells compared with other APCs such as DCs (80, 81).

In the context of healthy thymus, B cells can be found both in the thymic medulla and in the perivascular space (PVS). B cells populate the thymic medulla early in life as a result of the intrathymic maturation of B cell progenitors and these cells exert a pivotal role of APCs at the basis of central tolerance establishment (82). On the contrary, B cells populate the thymic PVS later in life, and they are composed of memory B cells and antibody-secreting plasma cells (83, 84). Therefore, similar to the bone marrow, the thymus may serve as a functional niche for antibody production. Yet, further studies are needed to confirm the role of the thymus in this context.

Notably, it has been reported that the thymus of myasthenic patients have several B cell follicles with active germinal centers (GCs) responsible for autoantibodies production (85). Similar to B cells in normal thymuses, B cells in the tumor tissue of TETs have been subdivided into two main populations, with one population confined mainly in the area of medullary differentiation, and a second population located within the PVS and organized in follicles with GCs (86). Indeed, the presence of GCs within thymomas was excluded by a more recent immunohistochemistry study that demonstrated GCs in peritumoral tissue but not within tumors of MG^+^ thymoma patients (87). Notably, in their study the authors reported that MG^+^ thymomas exhibit 3.75 times more ectopic GCs in peritumoral thymic tissue compared with MG^-^ thymomas; and the number of ectopic GCs positively correlated with a higher autoantibody titer, thus suggesting the involvement of these structures in the pathogenesis of MG. Further studies are required to explore the features and role of GCs in thymomas, in order to better understand the relation between these tumors and antibody-mediated autoimmune diseases.

B cells are less commonly observed in mixed and cortical thymomas, currently defined as type AB and B thymomas, suggesting that the area of cortical differentiation prevents B cell accumulation, thus limiting the antigen presentation and the anti-tumor activity of these cells in type AB and B thymomas (86). Moreover, a particular type of TETs defined as micronodular thymoma is known to be highly enriched in B cells and the tumor itself can promote monoclonal B cell expansion through the aberrant expression of chemokines such as CCL18 and CCL20, which interfere with normal B cell homeostasis (88). This altered B cell proliferation can lead to the development of intratumor lymphomas. Also TCs have been reported to be infiltrated by B cells, likely sustained by the loss of a well-organized thymic architecture with subsequent reduction of privileged sites such as areas of cortical differentiation (86). In TCs, a high density of CD20^+^ B cells has been recently associated with a favorable prognosis (89), possibly related to the APC function of B cells in activating anti-tumor CD8^+^ cytotoxic T cells in the TC microenvironment (90).

### 4.3 Dendritic cells

Apart from adaptive immune cells that can either originate from or infiltrate thymomas and TCs, also cells of the innate immune system, such as DCs, macrophages, and NK cells, can play a role in tumor progression and development of autoimmunity. Nonetheless, little is known about the role of these cells in TETs and in TET-associated ADs.

DCs are professional APCs with a crucial role in initiating and shaping immune responses by activating naïve T cells, driving T cell differentiation into effector lineages, and producing cytokines to control and regulate the ongoing immune response (91). Within the normal thymus, DCs are mostly located at the level of the thymic medulla, where they participate in the negative selection of thymocytes (92).

In TETs, two different studies reported the presence of mature DCs within thymomas by immunohistochemistry (93, 94). It should be noted, however, that in these studies thymoma-infiltrating DCs were identified on the basis of the expression of markers, such as the actin-bundling protein fascin and the calcium-binding protein S100 (93, 94), which are not expressed exclusively by DCs but shared with other cell types, suggesting that the use of recently implemented single-cell technologies that allow a precise identification and characterization of tissue DCs would significantly improve the ability to address the role of DCs in TETs. In any case, the studies demonstrated that fascin^+^ cells aggregate in clusters, and their amount correlates with clinical prognostic scores. In particular, Sato and colleagues reported a positive correlation between the number of fascin^+^ DCs and a favorable outcome for thymoma patients, and also demonstrated a paucity of DCs in TCs, overall indicating that DCs can activate anti-tumor immunity to suppress tumor growth and invasiveness (93).

Another study addressed the role of circulating DCs in TETs. In particular, Zhang and colleagues reported an interesting correlation between peripheral blood DCs and MG, comparing patients with MG- thymomas with patients affected by thymoma with ocular and generalized MG. They observed that the frequency of peripheral blood DCs decreased progressively with the increasing severity of the thymoma-associated AD. Indeed, patients with generalized MG showed lower DC counts compared with those with ocular MG, suggesting that the level of circulating DCs may be used as a marker to follow the progression of the TET-associated autoimmune condition (95).

### 4.4 Macrophages

Also macrophages, another myeloid immune cell population, have been reported to infiltrate TETs. Tumor-associated macrophages (TAMs) can be polarized into several subtypes, including the anti-tumor M1 and pro-tumorigenic M2 macrophages: while the former promote inflammation and anti-tumor responses, the latter are involved in pro-angiogenetic and wound-healing processes, downregulating the inflammation and favoring tumor progression (96). Omatsu and colleagues identified by immunohistochemistry two subsets of TAMs within TETs, characterized by the expression of CD68 and CD163 markers, respectively. They reported that the amount of CD68^+^ TAMs did not differ between thymomas and TCs, whereas the amount of CD163^+^ TAMs was higher in TCs compared with thymomas (94). Notably, CD163 is a marker commonly used to identify M2 macrophages (97). Therefore, the observations that the immune infiltrate of TCs is characterized by the abundancy of M2 macrophages combined with the paucity of DCs described above, all together suggest that TC microenvironment may hamper an efficient anti-tumor response. Accordingly, a recent study confirmed that the frequency of M2 macrophages in TC, identified by the expression of CD204, correlates with poor prognosis (91). Based on these observations, future targeted therapeutic approaches for TC patients should be aimed at inhibiting the differentiation of immune suppressive TAMs while promoting the differentiation of DCs able to promote antitumor immune responses (94).

### 4.5 NK cells

Another important player of innate immunity is represented by NK cells, which are involved in the immune surveillance against viral-infected and tumor-transformed cells. NK cells can directly kill target cells, but they can also modulate other immune cells by the secretion of cytokines and chemokines, and by regulating APCs (98). NK cells can be subdivided into two main subsets according to the expression on their membrane of CD56 and CD16: CD16^+^CD56^dim^ (CD56^dim^) NK cells and CD16^-^CD56^bright^ (CD56^bright^) NK cells (99). CD56^dim^ NK cells represent the vast majority of circulating NK cells and are endowed with potent cytotoxic activity, whereas CD56^bright^ NK cells are mainly responsible for the production of cytokines. To our knowledge, only one study investigated the frequency of tumor-infiltrating and circulating NK cells in patients with thymoma complicated with MG (100). Regarding tissue-infiltrating NK cells, it reported no differences in the frequency of the two major NK cell subsets comparing hyperplastic thymuses and thymomas with healthy thymuses. In contrast, the study demonstrated that the number of peripheral blood CD56^dim^ NK cells was significant lower in patients with MG^+^ thymoma compared with healthy controls. The authors hypothesized that the reduction of circulating CD56^dim^ NK cells in these patients may result from the recruitment of NK cells into the neuromuscular junction to perpetuate tissue damage (100).

## 5 Correlation between histotype and tumor microenvironment in thymic neoplasms

Traditionally, TETs have been subdivided into six categories, based on tumor features that include their malignancy, metastatic ability, and prognosis (2). The latest edition of WHO, 2021, 5th edition, distinguishes TETs as class A, AB, B1, B2, B3 or C (2, 101). Type A thymomas are characterized by a medullary phenotype, whereas B thymomas originate from the thymic cortex (101). Whereas the A, AB, B1, B2 histotypes are more differentiated and have a more favorable biological behavior, class C TETs includes thymic carcinomas (TCs), a group of very rare malignant neoplasms, generally with squamous differentiation, which are invasive and can metastasize (98). Finally, the B3 subtype is a neoplastic form midway between the more differentiated histotypes and TC (101, 102). Recently, there has been a specific interest in the study, characterization, and analysis of the tumor microenvironment (TME) of TETs, that can help us to understand whether, in addition to conventional chemotherapy, patients with thymic neoplasms could benefit from drugs such as immune checkpoint inhibitors (ICIs) (103–106).

For example, in a very recent paper Hou X. et al. reported that the different subtypes of TETs had different TME and genomic characteristics, which affected their biological behavior and prognosis. In particular, the authors performed a RNA-sequencing and whole exome sequencing (WES) on 21 TETs subdived into A, B1, B2, B3 and C histotypes (107). The authors first assessed the inflammatory and immune profile of tumors by comparing the immune and stromal scores among the various subtypes of TETs. They found no difference in the immune score between thymomas and TCs, except that the immune score was lower in the type A and TC. Conversely, in terms of stromal score, TCs exhibited the highest values among all thymoma subtypes, especially if compared to A, B1 and B2 histotypes. Then, the authors performed a single sample gene set enrichment analysis (ssGSEA) to calculate the enrichment scores (ES) of immune cells in the TME across different TET subtypes. Concerning the immune cell signatures, the authors identified 14 immune cell populations that were differently enriched in thymomas compared with TC, including activated CD8^+^ T cells, activated DCs, type 1 helper T cells and cells featuring tumor suppression functions such as immature DCs and myeloid-derived suppressor cells. The authors further reported a differential expression among different TET subtypes of several genes involved in the immune response, including CD40 and CD40-LG, TNFRSF14, IL-10, TGFB1, MICA and HMGB1. In particular, they demonstrated that, compared with type A and B thymomas, TCs expressed lower levels of HMGB1, a damage-associated molecule that is also secreted by activated immune cells and promotes the release of pro-inflammatory cytokines. Notably, a lower HMGB1 expression had a significant negative impact on the overall survival (OS) of the tested patients.

In the last few years, there has also been interest in evaluating the expression of the PDL-1/PD1 pathway in TME of TETs with a view to a possible application of immunotherapeutic agents. Some authors, such as Arbour K.C. et al., measured PD-L1 expression on tumor cells, but also on CD3^+^ and CD8^+^ tumor-infiltrating lymphocytes (TILs). In their paper, PD-L1 positivity resulted frequent in TETs, more common in thymomas (only B2 and B3 histotyoes we included) than TCs, and it was associated with longer OS than PD-L1 negative tumors (108). Similarly, Higuchi R. et al. reported that PD-L1 expression was higher in B2 and B3 thymomas than TCs (109). These data were argued by the authors as reflecting different pathogenesis, histopathology, and biological phenotype among types A, B, and C of TETs. The authors also reported that CD4^+^ and CD8^+^ T cells are more abundant in B2 and B3 thymomas and TC, compared to the remaining subtypes, paving the way for the possible use of ICIs as immunotherapy in TETs (4–8). Padda et al. analyzed 69 TETs and observed PD-L1 overexpression in 68% of cases (110).

Katsuya et al. analyzed 141 TET cases and reported high PD-L1 immunoexpression in 70% of TCs, but only in 23% of thymomas of different types (111). Finally, also Yokoyama et al. reported increased PD-L1 expression in 80% of TCs. It is important to consider how the variability of results among different studies may be related to intrinsic differences among the clones of antibodies used, the available instruments and the cut-off used to define a case as positive/negative (108–112).

In a very recent paper by Agrafiotis et al., the authors comprehensively reviewed the reliable data present in literature and summarized the current knowledge related to TME of TETs associated with a favorable or dismal prognosis (113). From these data, it was suggested that high expression of PD-L1 expression on tumor cells may be considered a marker of favorable prognosis, although this issue is controversial. Similarly, moderate/high levels of CD3^+^ TILs and a higher proportion of TILs, Th2 cells, CD8^+^ T cells, iDCs and neutrophils, appear to be predictive of a favorable prognosis. Conversely, a lower expression of HMGB1, a higher proportion of Tregs, NK cells, DCs and macrophages, together with a high tumor mutational burden, HSP27, HSP70 and SOX9 expression seem to be correlated with a dismal prognosis.

In conclusion, considering that the tumor micro-environment characteristics and its immuno-score represent novel potential prognostic and predictive biomarkers for cancer and its specific therapies, an in dept knowledge of them and their relationships with cancer development, immune system involvement, and autoimmune disorders is mandatory to improve clinical outcomes of patients.

## 6 Role of immunotherapy in thymic tumors with or without co-morbidities with ADs

### 6.1 Immune checkpoint inhibitors

Because of great levels of PD-L1 expression, ranging from 23% to 92% for thymomas and from 34% to 88% for TCs, PD-1/PD-L1 inhibitors have been tested in TETs showing a promising clinical activity. However, available data is currently limited to small phase I and II trials in advanced pretreated patients with refractory or recurrent disease (110, 111, 114, 115). **Table 2** summarizes the main trials.

**Table 2:** Immunotherapy in TETs.

Regimen	Author (year)	Stage	No. of pts	RR	mPFS (months)	mOS (months)
Thymomas						
**Pembrolizumab**	Cho et al. (20)	IV	7	29%	6.1	NR
**Avelumab**	Rajan et al. (116)	IV	7	29%	NA	NA
Thymic Carcinoma						
**Pembrolizumab**	Giaccone et al. (19)	IV	40	22.5%	4.2	24.9
	Cho et al. (20)	IV	26	19.2%	6.1	14.5
**Nivolumab**	Katsuya et al. (117)	IV	15	0% DCR 73.3%	3.8	14.1
**Avelumab**	Rajan et al. (116)	IV	1	0%	NA	NA
Thymomas and Thymic Carcinoma						
**Bintrafusp Alfa**	NCI (on going)	NA	38 (estimated)	NA	NA	NA

TETs, thymic epithelial tumors; No, number; Pts, patients; RR, response rate; mPFS, median progression free survival; mOS, median overall survival; NR, median not reached at time of data publication; NA, not available; DCR, disease control rate.

Cho et al. tested Pembrolizumab, an anti-PD-1 humanized monoclonal antibody, in a phase 2 trial enrolling 33 patients with advanced TETs, pretreated with at least one line of platinum-based chemotherapy (20). The primary end point was the overall response rate (ORR). Among 7 patients with thymoma and 26 patients with TC, the ORR was 28.6% (2 out of 7 patients) and 19.2% (5 out of 26 patients), respectively. Moreover, a stable disease (SD) was achieved in 71.4% of thymomas (5 out of 7 patients) and in 53.8% (14 out of 26 patients) of TCs. For both subgroups the median progression-free survival (PFS) was 6.1 months, while the median overall survival (OS) was not reached for patients with thymoma and was 14.5 months for patients with TC. It should be mentioned that 71.4% of patients with thymoma (5 out of 7 patients) discontinued immunotherapy due to the development of G3 or G4 immune-related adverse events (irAEs) (in particular, G4 myocarditis, hepatitis and glomerulonephritis and G3 thyroiditis and colitis). Moreover, among them, 4 patients fully recovered after immunosuppressive therapy including high doses of corticosteroids, while one patient died because of an opportunistic infection developed during the immunosuppressive treatment. On the other hand, only 11.6% of patients with TC (3 of 26 patients) experienced a G≥3 irAEs (G4 myasthenia and G3 autoimmune hepatitis and subacute myoclonus). All of them fully recovered after an adequate therapy.

In another phase 2 trial, Giaccone et al. evaluated Pembrolizumab in second and subsequent lines in 40 advanced TCs (19, 118). The primary end point was the ORR, reported in 22.5% of cases (1 complete response (CR) and 8 partial responses (PR), with a median duration of response (DOR) of 36 months, while a SD was observed in 21 patients (52.5%). Median PFS and OS were 4.2 and 24.9 months, respectively. Severe irAEs were reported in 6 patients (15%). The most common one was transaminitis. Two patients (5%) developed polymyositis and myocarditis, which required the placement of a pacemaker, as well as high doses of corticosteroids. All severe adverse events (AEs) resolved after immunosuppressive therapy.

In both studies mentioned above, the correlation between PD-L1 expression and response rate to Pembrolizumab was evaluated. High PD-L1 expression (positive staining in ≥50% of tumor cells) appears to be associated with increased overall survival and likelihood of response compared to absent or low PD-L1 expression (19, 20, 118). Obviously, larger prospective studies will be needed in the future to confirm these early findings on the role of PD-L1 expression as a predictive biomarker of response to Pembrolizumab in patients with TET. Furthermore, the available data mainly refer to patients with TC and the predictive role of PD-L1 expression in thymomas remains to be determined.

Nivolumab, another humanized monoclonal anti-PD-1 antibody, was tested in the PRIMER study (117). It was a single-arm, multicenter, phase II trial conducted on 15 patients affected by unresectable or recurrent TC, pretreated with at least one line of chemotherapy. Due to the inability of Nivolumab to provide at least one disease response, patients enrollment was interrupted at a preplanned futility interim analysis. However, a SD was observed in 73.3% of cases, with a median PFS of 3.8 months and a median OS of 14.1 months. In this trial 2 patients (13%) experienced serious irAEs including one G3 hepatitis and one G2 adrenal insufficiency, but no permanent discontinuation due to AEs occurred.

Avelumab, an anti-PD-L1 humanized monoclonal antibody, was evaluated in pretreated recurrent/refractory TETs. In a phase I dose-escalation study, it was tested on 8 patients (119). Among 7 thymomas, an ORR was observed in 4 patients (57.1%) and was confirmed in 2 of them with a repeat imaging (28.6%), while a SD and a progressive disease (PD) were reported in 2 (28.6%) and in 1 (14.3%) patient, respectively. The only enrolled patient with TC achieved a SD. All responders developed an immune-related toxicity (myositis and enteritis in 3 and one case, respectively), while only one of non-responders experienced it. In another still-ongoing phase II trial (NCT03076554) Avelumab has shown promising results: among 12 patients with thymoma and 10 patients with TC who were evaluable for response, it has been observed an ORR of 16.7% and 20%, a SD of 83.3% and 60%, respectively. The median PFS was 6.4 months in the thymoma subgroup and 14.7 months in the TC group (116).

Interesting data has been recently published about the efficacy of combining ICIs to tyrosine-kinase inhibitors (TKIs). Rationale of this strategy is a synergistic effect of these two types of drugs, as previously showed in several types of solid tumors (120, 121). In the CAVEATT trial, a single-arm phase 2 study, the combination of Avelumab with Axitinib was evaluated as second and subsequent lines of treatment in 32 patients (27 with TC, 3 with type B3 thymoma and 2 with a mixed type B3 thymoma and TC). The primary end point of this trial was ORR. The ORR was observed in 11 (34%) out of 32 enrolled patients, with a median DOR of 5.5 months. Stable disease was observed in 18 patients (56%). Median PFS was 7.5 months and median OS was 26.6 months. The most frequent G3-4 AE was hypertension, related to Axitinib. Grade 3-4 irAEs occurred in 4 (12%) patients (2 cases of G3 polymyositis, one case of G3 interstitial pneumonitis, and one case of G4 polymyositis), but there were no treatment-related deaths. In this trial, no correlation between PD-L1 expression status by immunohistochemistry (staining defined as high: ≥50%, low: 1–49% or negative: <1%) and ORR or PFS was observed. This observation could be explained by the synergistic effect obtained with the combination of a TKI and a PD-L1 inhibitor (122).

Several others ongoing trials are investigating ICIs in TETs alone or in combinations with different agents, as chemotherapy (NCT03134118 and NCT03858582 trials), multi-target TKI (sunitinib in NCT03463460 trial; lenvatinib in NCT04710628 trial, vorolanib in NCT03583086 trial), other ICIs (ipilimumab in the cohort 2 of NIVOTHYM trial and KN046, a bi-specific antibody against PD1 and CTLA 4, in the NCT04925947 phase II trial), and immunomodulatory agents. Indoleamine 2,3-dioxygenase-1 (IDO1), is a catalytic enzyme that contributes to T-cell response suppression. Pembrolizumab plus Epacadostat, an IDO1 inhibitor, is being evaluated in 45 pre-treated TET patients in an ongoing phase II clinical trial (NCT02364076). This study showed interesting preliminary results, with an ORR of 22.5%, a DCR of 75%, a median PFS of 4.2 months and OS of 24.9 months (123). Bintrafusp alfa is a bifunctional fusion protein against TGF-β and PD-L1, being tested in TET patients progressed after at least one platinum-containing chemotherapy in an ongoing phase II trial (NCT04417660) (124).

The potential benefits of ICIs in TETs should be carefully balanced with an increased risk of immune toxicity, because of a higher rate of irAEs than observed in most other malignancies, despite the exclusion from all the above-mentioned clinical trials of patients with a history of autoimmune disease. A higher percentage of potentially severe irAEs was observed in particular for patients affected by thymoma, while patients affected by TC seem to be less susceptible to this type of toxicity (20, 125). The most frequently reported G3–4 irAEs in TET trials included transaminitis, myalgia, myositis, enteritis, myocarditis, thyroiditis, nephritis and colitis (19, 20, 117, 119); a simultaneous occurrence of multiple adverse events can also occur (126). Moreover, patients with TET treated with ICIs have shown a higher predisposition to develop musculoskeletal, neuromuscular and cardiac autoimmune toxicity if compared to other malignances, where a median incidence <1% is reported (127–129).

Interestingly, the development of immune toxicity has been associated with the response, as observed in other types of cancer (113, 114, 130–133).

The reasons for the increased incidence of irAEs in TET patients undergoing ICI therapy are not fully known. Possible explanations can be found not only in the immune role of the thymus and in the particular predisposition to immune paraneoplastic syndromes observed in patients with thymoma, but also in the combined effects of anti-PD-1/PD-L1 drugs on this substrate (134, 135). In peripheral lymphoid organs, the PD-1/PD-L1 interaction has a fundamental role in the normal generation of immune tolerance and it is possible that ICIs may hinder the death process of thymic epithelial cells, causing a loss of immunotolerance. and therefore favoring the development of irAE (136).

These findings impose to closely monitor patients with TC receiving ICIs and to avoid ICIs for thymoma treatment outside of clinical trials, while currently National Comprehensive Cancer Network (NCCN) guidelines consider Pembrolizumab as a second-line treatment option for TC (5).

### 6.2 Cancer vaccines

Cancer vaccines represent another pillar of immunotherapy in solid tumors. They aim to stimulate anti-tumor immune responses through T-cell activation against specific tumor-associated antigens (TAAs) or neoantigens. Indeed, TETs usually show a low tumor mutational burden (TMB), resulting in a poor expression of these antigens. As a consequence of this, only a few potential targets for cancer vaccines to be used in TET patients can be identified (137). Accordingly, literature on this topic is scarce, with only one prospective study available reporting the use of a WT-1 peptide vaccine. It was studied in a phase II trial enrolling patients with relapsed/refractory thymoma and TC expressing WT-1 protein, which was shown to be overexpressed in the majority (≥ 80%) of TET patients (132). Among the evaluable patients, no disease response was observed, but 6 out of 8 TC patients (75%) and 3 out of 4 thymoma patients (75%) obtained a SD. AEs related to WT1 vaccination were infrequent, with the exception for G1 erythema and swelling, which occurred in all treated patients. After more than 2 years of therapy, 2 patients affected by thymoma experienced irAEs, including one case of G3 pure red cell aplasia and one case of G2 myasthenia gravis. These results suggest a potential clinical activity of WT1 peptide vaccine for TETs, with an apparently acceptable safety profile in TC patients, but further investigation may be needed about irAEs in thymoma patients (138).

## 7 Discussion

Understanding the role(s) of immune responses in the pathogenesis of TETs is highly relevant as, under homeostatic condition, the thymus plays a pivotal role for T cell differentiation and for the selection of autoreactive T cells ensuring an appropriate tolerance against “self”. This functional feature of thymus mainly occurs in the first years of life, while decades in aging with the fibrotic involution of the organ.

The use of ICIs represents a promising therapeutic option for TETs as reported for the administration of anti-PD-1 biologic drugs. However, we do not know which immune cells express PD-1 and whether the IC blocking occurs with the same efficiency within the TME and PB anatomic compartments of TET patients. The paucity of information about immunopathogenesis of TETs is worsened by the rarity of these diseases. Indeed, the present clinical recommendations primarily rely on retrospective evaluations, non-randomized prospective studies, or expert consensus. Therefore, the clinical management of TETs is still very challenging and highly limited in our knowledge to possibly re-program or modulate immune cell functions by administering novel anti-tumor immune-therapeutic drugs.

Nonetheless, another major clinical challenge of TETs in human adults is represented by their co-morbidities with ADs. Specifically, B1 and B2 Thymoma histological subtypes are mainly associated with MG in 44% of affected patients. Conversely, patients with TC seldom experience the onset of autoimmune diseases (139). The pathophysiological links between TETs and autoimmunity as well as the dichotomy between Thymoma and TC in their co-morbidity with ADs are completely unknown. We cannot even stratify whether the presence of ADs worsens the prognosis and the clinical outcomes of TETs or vice versa. Indeed, while a pre-existing autoimmune condition does not constitute a risk for AD relapse after surgery, the impact of surgical resection on AD flares is unpredictable (140). Several theories had been postulated to explain the pathogenic mechanisms associating TETs and AD (139, 141). These working hypotheses speculate on possible failures of negative thymic selection of T-lymphocytes in adulthood, although there are no experimental evidence supporting such postulates. Moreover, while NK cells and B lymphocytes as well as of DCs have been described to play a major role in the pathogenesis of several solid cancers and ADs, very little is known about their impact on TET onset either associated or not with autoimmunity. Not even a clonal expansion of potential autoreactive T and B cell clones had never been reported in TET patients. In this scenario, our incapacity to identify Thymoma patients at higher risk of developing ADs together with the lack of guidelines in clinical monitoring and therapies represents an important unmet clinical need. In fact, the association between TETs and ADs makes even more difficult the administration of those novel immunotherapies targeting several immune inhibitory checkpoints, due to the higher risk of exacerbating autoimmunity in these patients. In fact, if immunotherapy with anti-PD-1 inhibitors is a promising therapeutic option for TETs, the identification of biomarkers able to predict AD development would be crucial to select patients in which the beneficial effects of immune checkpoint blockade would be counteracted by severe immune-related side effects characterized by activation/recrudescence of autoimmune disorders, that may potentially result fatal (19, 20). Indeed, our lack of knowledge of the immunologic mechanisms disrupted within the TME of TETs greatly limits our ability to predict and control cancer progression. Therefore, an in-depth exploitation of the immune pathogenesis of TETs and associated autoimmunity is key to better predict patients’ clinical outcomes and to improve their therapeutic options. In fact, physician needs to know why some patients develop AD after thymectomy or vice versa or if there are patients at higher risk to develop ADs. Indeed, their monitoring and clinical follow up is still very challenging, have high social costs and lack clear/shared guidelines in the presence of life-threatening events. Hence, a better understanding of the pathophysiological links between thymomas and autoimmunity and the identification of potential predictive biomarkers to develop ADs could allow physicians to: a) select patients at higher risk of developing autoimmune disorders and provide specific clinical monitoring only to this specific and recognizable cohort of TET’s patients; b) possibly prevent the onset of ADs either with preventive surgery or *ad hoc* therapies targeting a specific immune cell compartment or ICs. This would reduce the impact of the economic and social costs necessary for their treatment and, most importantly, would improve the quality of life of these patients; c) avoid the immune-related side effects or the activation/recrudescence of autoimmune disorders induced by immunotherapies that represent promising therapeutic options for thymomas.

In conclusion, TETs represent one of the most interesting disease models at the intersection between tumor-immunology and autoimmunity. However, our knowledge of the biological basis regulating the relationships among immune system, TETs, and systemic autoimmune diseases still remains scarce and obscure. Therefore, if in the clinical setting a multidisciplinary approach is required to improve outcomes and reduce long-term treatment-related side effects, also to improve our understanding of the immune pathogenesis of TETs and associated autoimmunity will need a multidisciplinary research approach, favoring the collaboration among professionals as the immunologist, oncologist and pathologist.

## Author contributions

MP: Conceptualization, Data curation, Writing – original draft, Writing – review & editing. EV: Conceptualization, Data curation, Writing – original draft, Writing – review & editing. SB: Data curation, Writing – original draft, Writing – review & editing. GC: Data curation, Writing – review & editing. EF: Data curation, Writing – review & editing. SF: Data curation, Writing – review & editing. MD: Resources, Writing – review & editing. FDV: Resources, Writing – review & editing. NC: Resources, Writing – review & editing. RT: Resources, Writing – review & editing. FB: Resources, Writing – review & editing. MAli: Resources, Writing – review & editing. MAi: Resources, Writing – review & editing. LGC: Resources, Writing – review & editing. MAll: Data curation, Writing – review & editing. AS: Data curation, Writing – review & editing. LDT: Data curation, Resources, Visualization, Writing – review & editing. GI: Data curation, Writing – original draft. AV: Data curation, Writing – original draft. SDB: Data curation, Writing – original draft, Writing – review & editing. PAZ: Data curation, Methodology, Resources, Writing – original draft. DM: Resources, Writing – original draft, Writing – review & editing. RF: Data curation, Writing – review & editing. GM: Data curation, Writing – review & editing. LR: Data curation, Writing – review & editing. GDR: Data curation, Writing – review & editing.
